# The Impact of Tube-Shaped Bladder Neck Modification on Early-Term Continence in Robotic Radical Prostatectomy

**DOI:** 10.5152/tud.2026.26053

**Published:** 2026-08-19

**Authors:** Yasin Aktaş, Kayhan Yılmaz, Şahin Kılıç, Murat Şambel, Mahmut Taha Ölçücü, Mutlu Ateş

**Affiliations:** 1 Department of Urology, Uşak University Faculty of Medicine, Uşak, Türkiye; 2 Department of Urology, Health Sciences University Antalya Training and Research Hospital, Antalya, Türkiye

**Keywords:** Prostate cancer, robotic radical prostatectomy, continence, modified urethral anastomosis technique

## Abstract

**Objective:**

We aimed to investigate the effect of tube-shaped bladder neck, which we define as our modified urethral anastomosis technique, on early continence in patients undergoing robot-assisted radical prostatectomy.

**Methods:**

This retrospective study (2017-2020) compared standard anastomosis (group 1, n = 75) with a modified technique (group 2, n = 74) in patients undergoing radical prostatectomy. Preoperative, perioperative, postoperative, and histopathological characteristics of the patients were recorded. Continence was assessed using the Turkish version of the International Consultation on Incontinence Questionnaire Short Form (ICIQ-SF), and daily pad usage was recorded. Patients who never used a pad and had an ICIQ-SF score of ≤6 were considered continent. Erectile function was evaluated using the International Index of Erectile Function-5 form.

**Results:**

When evaluating postoperative continence status, the number of pads used and the ICIQ-SF score were significantly lower in the group that received the new technique at the first, third, and sixth months. In our study, the postoperative continence rates were 18.7%-36.5% at 1 month, 38.7%-63.5% at 3 months, and 56%-77% at 6 months for groups 1 and 2. Our new technique provided statistically superior results during the first 6 months (*P* = .015, *P* = .002, and *P* = .007, respectively).

**Conclusion:**

The modified urethral anastomosis technique accelerates the recovery of early continence (within 1-6 months), while achieving comparable long-term outcomes to those of the standard method. Our new technique is oncologically reliable and does not compromise cancer control.

**Main Points**

- The tube-shaped bladder neck modification is a novel urethral anastomosis technique that significantly accelerates the recovery of early urinary continence following robot-assisted radical prostatectomy, as evidenced by statistically lower daily pad usage and improved ICIQ-SF scores within the first 6 months compared with the standard method.
- In addition to its significant early advantages, the modified technique achieves comparable long-term continence outcomes to the standard approach at the 12-month follow-up.
- The anatomical modification is an oncologically safe procedure that demonstrates comparable surgical margin positivity rates to the standard anastomosis without compromising cancer control.

## Introduction

Prostate cancer is the most commonly diagnosed cancer in men in European countries.^1^ Radical prostatectomy (RP) is often used to treat local and locally advanced disease. This procedure can be performed in an open, laparoscopic, or robotic-assisted approach. Recent evidence highlights that robot-assisted radical prostatectomy (RARP) offers acceptable oncological and patient-reported outcomes, even in frail or selected elderly populations, and provides advantages such as shorter hospital stays and lower transfusion rates compared with open surgery.^2^ Furthermore, the enhanced 3-dimensional visualization and manoeuvrability provided by RARP allow anatomical and functional structures, such as the external urethral sphincter and bladder neck, that contribute to urinary continence to be more easily preserved compared with open surgery.^3^ However, as with retropubic radical prostatectomy (RRP) and laparoscopic radical prostatectomy (LRP), an important complication of RARP is urinary incontinence (UI), which negatively impacts patients’ quality of life and psychological well-being, regardless of the results regarding oncological and sexual function.^3^

In a systematic review by Basiri et al, which included 104 research articles comparing the oncological and functional results of RRP, LRP, and RARP, RARP was interpreted as more successful in terms of postoperative UI and erectile dysfunction (ED).^4^ Nevertheless, the establishment of urinary continence following RP remains a challenge to overcome.^5^

Technical modifications, such as bladder neck preservation, neurovascular bundle preservation, bladder neck repair, and support, have been shown to reduce UI rates after RP.^6^ These techniques typically aim to recreate the normal anatomy of the bladder neck, vesicourethral anastomosis, urethral sphincter, and urethra, as well as the surrounding tissue, while preserving their functions. Based on this, Freire et al studied anatomic bladder neck preservation in patients who underwent RARP.^7^ In Rocco et al’s posterior reconstruction technique for RP, the vesicourethral anastomosis is supported from behind by suturing the Denonvilliers fascia and the inferior part of the external sphincter together (the Rocco suture).^8^ It was concluded that this technique accelerates the return to continence in the short term.^9^

Historically, foundational techniques, such as the Heilbronn ascending approach, have introduced alternative strategies for managing the bladder neck and pelvic anatomy during LRP.^10^ Evolving from these early principles of bladder neck management, and supported by contemporary evidence indicating that specific tubulization and anterior reconstruction techniques may facilitate early continence recovery,^11,12^ we implemented a modified anastomosis approach. We hypothesized that applying a bidirectional continuous suture to create a tube-shaped narrowing 2-3 cm proximal to the bladder neck could decrease the pressure transmitted to the external sphincter while simultaneously aiding haemostasis. Thus, we investigated the effect of this modified technique on early continence in patients undergoing RARP.

## Material and Methods

Approval was obtained from the Ethics Committee of the Health Sciences University Antalya Training and Research Hospital prior to the study (Decision No. 9/5, Date: June 18, 2020).

We retrospectively scanned the data of patients who underwent RARP due to prostate cancer in the urology clinic of Health Sciences University Antalya Training and Research Hospital between September 1, 2017, and January 15, 2020. The postoperative data of the patients were recorded.

Our research was designed in accordance with the Declaration of Helsinki and the principles of Good Clinical Practice, and it did not conflict with the ethical rules of subject research.

The study included patients diagnosed with prostate cancer in our clinic who accepted surgery and were scheduled for a RP. Of those patients, 149 who chose the robotic method were included in the study. We applied our standard anastomosis method to 75 patients and our modified urethral anastomosis technique to the remaining 74 patients. Patients were included in the study consecutively. The study groups were formed based on a chronological timeline reflecting the evolution of the surgical technique. Group 1 consisted of patients operated on between 2017 and 2019, while group 2 comprised patients operated on from 2019 to 2020, following the institution’s adoption of the modified technique. The operations were performed by a single surgeon who had an experience of at least 250 RARP procedures prior to the 1 patient included in the standard group. If there was no oncological contraindication, nerve-sparing RP was performed on all patients with normal erections. Extended pelvic lymph node dissection (ePLND) was performed on patients with intermediate- or high-risk disease, specifically those with a risk of nodal metastasis greater than 5% based on the Briganti nomogram.

Patients with missing data, high intracranial pressure, or serious lung or heart problems or those who would be at serious risk if they remained in the Trendelenburg position for a long time were not suitable for robotic surgery and were excluded from the study. Patients who received hormonal therapy and adjuvant radiotherapy were also excluded, and consequently, patients with a clinical stage of >cT2c were naturally eliminated from the cohort, as they typically receive these treatments in our daily clinical practice. Additionally, patients who underwent transurethral resection of the prostate (TURP), had a history of urethral stricture, or experienced UI were excluded from the study.

The demographic data of patients were recorded. The International Index of Erectile Function-5 (IIEF-5) questionnaire was used to assess erectile status before the operation. Postoperative complications were classified according to the Clavien–Dindo classification.^13^ We applied a standard institutional protocol for pelvic floor muscle training. All patients in both groups were explicitly taught these exercises and advised to perform them after surgery.

The pathology specimen results and blood prostate-specific antigen values of patients who were called for outpatient clinic control 1 month after surgery were recorded. Continence was assessed using the Turkish version of the International Consultation on Incontinence Questionnaire Short Form (ICIQ-SF), and daily pad use was recorded.^14^ Patients who did not use any pads or used 1 safety pad and had an ICIQ-SF score of ≤6 were considered continent. Later, patients who came for routine prostate-specific antigen control at 3, 6, and 12 months were asked to complete the aforementioned questionnaire again.

This study used generative AI/LLM tools (specifically, Gemini) to improve language and edit grammar during the preparation of the manuscript. However, the authors reviewed and edited all content following the use of AI/LLM tools.

### Statistical Analysis

The IBM SPSS Statistics 23 program (IBM Corp., Armonk, NY, USA) was used to analyse the data. Descriptive statistics are presented as numbers (%), mean ± standard deviation, and median (interquartile range). The Pearson chi-square test and Fisher’s exact test were used to examine the relationships between the categorical variables. The Shapiro–Wilk test was used to check the normality assumption. The Mann–Whitney *U* test analysed differences between the measurements of the 2 groups with a skewed distribution, while Student’s *t* test was used for normally distributed data. Friedman’s test analysed changes over time when the data were not normally distributed. Multivariate logistic regression analysis was performed to identify continence-affecting factors in patients, using factors yielding a *P* value of <.1 in univariate analysis. The results are presented as odds ratios and 95% confidence intervals (CIs). *P* values less than .05 were considered significant.

### Surgical Technique

#### Tube-Shaped Bladder Neck Technique

The surgeries were performed with the patient under general anaesthesia and in a 30° Trendelenburg position. Standard surgical preparation was performed with a transperitoneal anterior approach. The da Vinci Xi (Intuitive Surgical, Sunnyvale, CA, USA) system was used for all surgeries. The surgical steps up to the vesicourethral anastomosis were performed in the order described in the literature for RARP with a transperitoneal anterior approach.^5^ In our clinic, we routinely apply the techniques of bladder neck preservation, bladder neck–urethra approximation (posterior reconstruction), and ligation of the dorsal vein complex and suspension to the pubis (anterior reconstruction) in RP surgeries. After completing the surgical steps described, the bladder neck was identified. To narrow the bladder neck, 2-0 multifilament absorbable continuous sutures were applied at 1-cm intervals, starting approximately 2 cm proximal to the bladder neck. This resulted in a tubular narrowed bladder neck. The sutures were advanced through the serosal and muscular surfaces of the bladder, with an aim to remain on the serosal surface as much as possible. Suturing began on the right lateral side and continued at 1-cm intervals until reaching the left lateral side. The suturing then continued by returning 1 cm proximal to the left lateral side, and the procedure was terminated by tying the threads on the right lateral side (Figure 1).^15^

**Figure 1.**
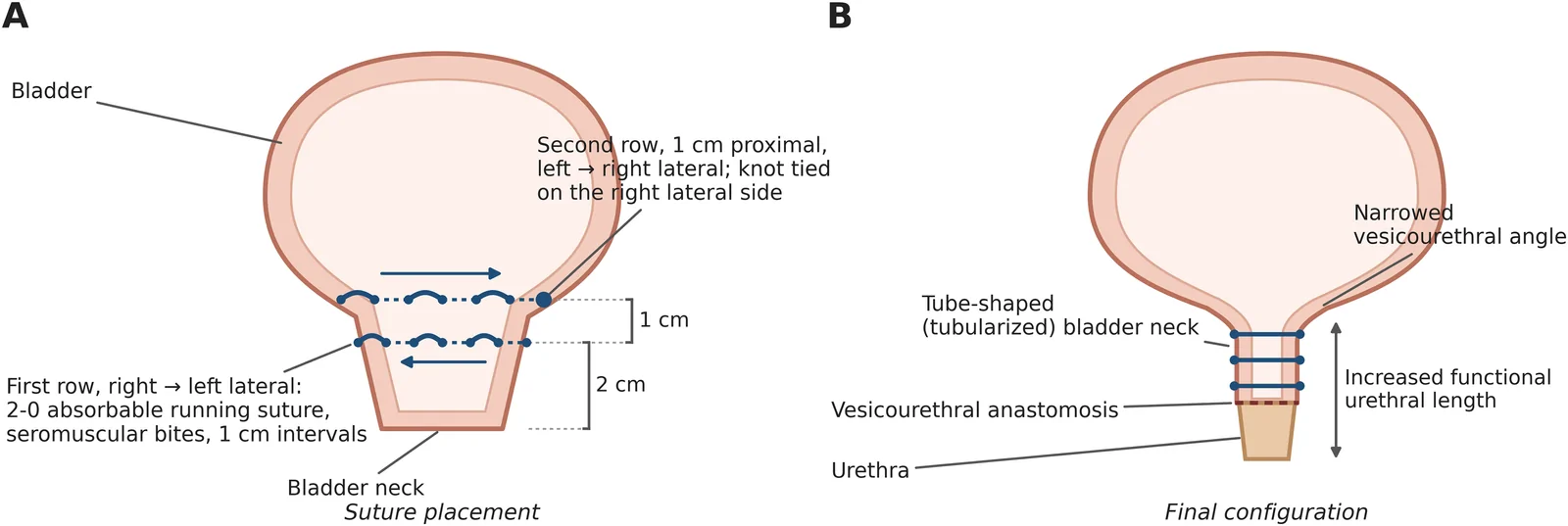
Schematic illustration of the modified tube-shaped bladder neck technique. (A) Bidirectional double-row suture placement. (B) Final anatomical configuration demonstrating the tubularized bladder neck prior to vesicourethral anastomosis.

The aim was to narrow the angle of the vesicourethral junction and partially resemble the geometry of the bladder neck to that of the urethra, thereby increasing the functional urethral length. Reflux bleeding was prevented while also aiming to reduce the reflection of intra-bladder pressure to the external urethral sphincter in cases of increased intra-abdominal pressure.

## Results

A total of 149 patients who underwent RARP for prostate cancer between September 2017 and January 2020 were included in the study. We applied our routine anastomosis technique to 75 patients (group 1) and our modified urethral anastomosis technique to the remaining 74 patients (group 2). The preoperative characteristics of the patients are summarized in Table 1.

**Table 1.** Preoperative Characteristics of Patients

Variables	All Patients (n = 149)	Group 1 (n = 75)	Group 2 (n = 74)	*P* Values
Age	63.99 ± 5.53	64.17 ± 5.63	63.8 ± 5.45	.679
BMI	27.63 ± 3.42	27.42 ± 3.09	27.86 ± 3.73	.434
Additional diseases				
DM	4 (2.7)	0 (0)	4 (5.4)	.058
HT	60 (40.3)	39 (52)	21 (28.4)	**.003**
ASCVD	25 (16.8)	13 (17.3)	12 (16.2)	.855
COPD	11 (7.4)	7 (9.3)	4 (5.4)	.359
Neurological	7 (4.7)	2 (2.7)	5 (6.8)	.276
IIEF total score	17(12-20)	17 (11-21)	15.5 (12-20)	.329
IIEF level				
Severe ED	14 (9.4)	10 (13.3)^a^	4 (5.4)^a^	**.024**
Moderate ED	19 (12.8)	8 (10.7)^a^	11 (14.9)^a^	
Mild–moderate ED	39 (26.2)	12 (16)^a^	27 (36.5)^b^	
Mild ED	48 (32.2)	27 (36)^a^	21 (28.4)^a^	
No ED	29 (19.5)	18 (24)^a^	11 (14.9)^a^	
Prostate volume (cc)	47 (35-60)	45 (40-60)	50 (35-65)	.976
PSA	8 (5.76-12)	7.61 (5.54-11)	8.27 (6-14)	.209
Haemoglobin	14.9 (13.8-15.6)	14.7 (14-15.4)	14.95 (13.6-15.7)	.745
Gleason total	6 (6-7)	6 (6-7)	7 (6-7)	.115
ISUP				
3 + 3	79 (53)	45 (60)	34 (45.9)	.378
3 + 4	39 (26.2)	19 (25.3)	20 (27)	
4 + 3	14 (9.4)	5 (6.7)	9 (12.2)	
3 + 5 or 4 + 4	13 (8.7)	5 (6.7)	8 (10.8)	
≥9	4 (2.7)	1(1.3)	3 (4.1)	
Clinical stage				
T1a	1 (0.7)	1 (1.3)	0 (0)	.154
T1c	102 (68.5)	51 (68)	51 (68.9)	
T2a	19 (12.8)	13 (17.3)	6 (8.1)	
T2b	5 (3.4)	1 (1.3)	4 (5.4)	
T2c	22 (14.8)	8 (12)	13 (17.6)	
D’Amico risk				
Low	55 (36.9)	30 (40)	25 (33.8)	.640
Medium	58 (38.9)	29 (38.7)	29 (39.2)	
High	36 (24.2)	16 (21.3)	20 (27)	

Note: The results are presented as mean ± standard deviation, median (interquartile range), or number (%). Student’s *t* test, the Mann–Whitney *U* test, Pearson’s chi-square test, and Fisher’s exact test were used. Different lowercase letters in a row indicate a statistically significant difference between groups. Bold values indicate statistical significance (*P* < .05).

ASCVD, atherosclerotic cardiovascular disease; BMI, body mass index; COPD, chronic obstructive pulmonary disease; DM, diabetes mellitus; ED, erectile dysfunction; HT, hypertension; IIEF, the International Index of Erectile Function-5; ISUP, the International Society of Urological Pathology; PSA, prostate-specific antigen.

A significantly higher rate of lymph node dissection (LND) was observed in group 2 compared with group 1 (51.4% vs 33.3%, *P* = .026). The median anastomosis time was 20 minutes in group 1 and 30 minutes in group 2. Group 2 had a longer anastomosis time (*P* < .001). Perioperative and postoperative characteristics of the patients are summarized in Table 2. Histopathological characteristics of the patients are shown in Table 3.

**Table 2.** Perioperative and Postoperative Characteristics of Patients

Variables	All Patients (n = 149)	Group 1 (n = 75)	Group 2 (n = 74)	*P* Values
Lymph node dissection	63 (42.3)	25 (33.3)	38 (51.4)	**.026**
Major vascular complications	2 (1.3)	0 (0)	2 (2.7)	.245
Organ injury during surgery	6 (4)	0 (0)	6 (8.1)	**.013**
Nerve protection				
Not protected	53 (35.6)	28 (37.3)	25 (33.8)	.448
Unilaterally protected	30 (20.1)	12 (16)	18 (24.3)	
Bilaterally protected	66 (44.3)	35 (46.7)	31 (41.9)	
Anastomosis time (min)	30 (20-35)	20 (20-30)	30 (25-40)	**<.001**
Blood loss (cc)	200 (150-250)	200 (150-250)	200 (200-250)	.800
Operation time (min)	210 (180-262.5)	180 (150-240)	240 (180-300)	**<.001**
Haemoglobin	13.4 (12.3-14.2)	13.3 (12.5-14.2)	13.4 (12.1-14.1)	.745
Delta haemoglobin	1.3 (0.9-2)	1.3 (0.9-1.8)	1.3 (0.8-2.2)	.802
Drain removal (d)	3 (2-3)	3 (3-3)	3 (2-3)	.054
Discharge (d)	4 (3-4)	4 (3-4)	4 (3-4)	.428
Postoperative Clavien degree				
None	128 (85.9)	69 (92)^a^	59 (79.7)^b^	**.008**
1	14 (9.4)	2 (2.7)^a^	12 (16.2)^b^	
2	4 (2.7)	2 (2.7)^a^	2 (2.7)^a^	
3a	2 (1.3)	2 (2.7)^a^	0 (0)^a^	
3b	1 (0.7)	0 (0)^a^	1 (1.4)^a^	
Catheterization duration (d)	9 (8-10)	9 (9-10)	8 (8-9)	**<.001**

Note: The results are presented as median (interquartile range) or number (%). The Mann–Whitney *U* test, Pearson’s chi-square test, and Fisher’s exact test were used. Different lowercase letters in a row indicate a statistically significant difference between groups. Bold values indicate statistical significance (*P* < .05).

**Table 3.** Histopathological Characteristics of Patients

Variables	All Patients (n = 149)	Group 1 (n = 75)	Group 2 (n = 74)	*P* Values
Specimen weight (g)	48 (38-66)	50 (40-65)	47 (35-68)	.655
Gleason total	7 (6-7)	7 (6-7)	7 (6-7)	.165
International Society of Urological Pathology				
3 + 3	54 (36.5)	30 (40.5)	24 (32.4)	.639
3 + 4	46 (31.1)	20 (27)	26 (35.1)	
4 + 3	24 (16.1)	14 (18.6)	10 (13.5)	
3 + 5 or 4 + 4	12 (8.1)	6 (8.1)	6 (8.1)	
≥9	13 (8.8)	5 (6.8)	8 (10.8)	
Pathological stage				
T2	3 (2)	3 (4.1)	0 (0)	.406
T2a	19 (12.8)	10 (13.5)	9 (12.2)	
T2b	17 (11.5)	7 (9.5)	10 (13.5)	
T2c	52 (35.1)	28 (37.8)	24 (32.4)	
T3a	36 (24.3)	18 (24.3)	18 (24.3)	
T3b	21 (14.2)	8 (10.8)	13 (17.6)	
Extraprostatic spread	54 (36.5)	24 (32.4)	30 (40.5)	.306
Lymphovascular invasion	31 (20.9)	14 (18.9)	17 (23)	.545
Perineural invasion	116 (78.4)	55 (74.3)	61 (82.4)	.231
Seminal vesicle invasion	21 (14.2)	8 (10.8)	13 (17.6)	.239
Surgical margin positivity	35 (23.6)	17 (23)	18 (24.3)	.847
Lymph node positivity	8 (12.7)	0 (0)	8 (21.1)	**.018**

Note: The results are presented as median (interquartile range) or number (%). The Mann–Whitney *U* test, Pearson’s chi-square test, and Fisher’s exact test were used. Bold values indicate statistical significance (*P* < .05).

Significant differences were found between the 2 groups when evaluating the patients’ postoperative continence status according to the number of pads used at the first, third, and sixth months (*P* < .001, *P* < .001, and *P* = .005, respectively). Similarly, a significant difference was found between the 2 groups at the first, third, and sixth months according to the ICIQ-SF score, which evaluates continence status. Based on these results, our new technique appears to be superior to the standard technique in promoting continence within the first 6 months. However, no significant difference was found between the 2 groups at the 12-month follow-up. The number of pads and ICIQ-SF scores of the patients at various times according to the study groups are shown in Table 4.

**Table 4.** Number of Pads and ICIQ-SF Scores of Patients at Various Times According to Study Groups

Variables	All Patients	Group 1	Group 2	*P* Value^a^
Number of Pads				
First month	1 (0-3)^c^	3 (1-3)^c^	1 (0-2)^c^	**<.001**
Third month	0 (0-2)^d^	1 (0-2)^d^	0 (0-1)^d^	**<.001**
Sixth month	0 (0-1)^e^	0 (0-1)^e^	0 (0-0)^d,e^	**.005**
Twelfth month	0 (0-0)^e^	0 (0-0)^e^	0 (0-0)^e^	.208
*P*^b^	**<.001**	**<.001**	**<.001**	
ICIQ-SF Score				
First month	7 (3-12)^c^	11 (5-13)^c^	6 (1-11)^c^	**<.001**
Third month	3 (1-8)^d^	5 (2-10)^d^	1 (1-5)^d^	**<.001**
Sixth month	0 (0-4)^e^	0 (0-5)^e^	0 (0-0)^e^	**.004**
Twelfth month	0 (0-0)^e^	0 (0-0)^e^	0 (0-0)^e^	.253
*P*^b^	**<.001**	**<.001**	**<.001**	

Note: The results are presented as median (interquartile range). Different lowercase letters in a column indicate a statistically significant difference between groups. Bold values indicate statistical significance (*P* < .05).

a The Mann–Whitney *U* test.

b The Friedman test.

ICIQ-SF, International Consultation on Incontinence Questionnaire Short Form.

Univariate and multivariable logistic regression analyses were performed at 1, 3, and 6 months to identify independent predictors of early continence recovery. Because a significantly higher prevalence of hypertension (HT) was observed in group 1 at baseline (52% vs 28.4%, *P* = .003), it was critical to evaluate HT as a potential confounding factor. At the 1-month follow-up, the multivariable analysis revealed that the modified technique (odds ratio [OR]: 2.987, 95% CI: 1.369-6.515, *P* = .006) and the preoperative Gleason score were independent predictors of continence. At the 3-month follow-up, potential confounders, including HT, were incorporated into the multivariable model. The results showed that the modified technique (OR: 3.308, 95% CI: 1.550-7.061, *P* = .002) and positive surgical margins (*P* = .033) were independent predictors, but HT did not reach statistical significance. Similarly, at 6 months, although HT approached statistical significance in the univariate analysis (*P* = .053) and was included in the model, the modified technique remained the primary independent predictor of urinary continence (OR: 2.744, 95% CI: 1.232-6.110, *P* = .013). This confirms that the surgical technique is associated with improved continence outcomes, regardless of HT. When comparing baseline sexual function, there was no statistically significant difference in preoperative IIEF total scores between the groups (median: 17 vs 15.5, *P* = .329). However, a significant difference was observed in the ‘mild–moderate ED’ category specifically (*P* = .024), and this subgroup variation was analysed in our regression models. Multivariate analysis showed that baseline ED severity did not have a statistically significant effect on early continence recovery at any time point.

Potential confounders, such as body mass index (*P* = .935, *P* = .230, and *P* = .948, respectively), prostate volume (*P* = .508, *P* = .475, and *P* = .561, respectively), and bilateral nerve-sparing status (*P* = .268, *P* = .135, and *P* = .099, respectively), were evaluated at 1, 3, and 6 months. As none of these variables were found to be statistically significant predictors of continence outcomes (*P* > .05 for all time points), they were not included in the multivariable models.

Univariate analysis revealed that LND was not a statistically significant predictor of urinary continence at 1, 3, and 6 months (*P* = .620, .707, and .763, respectively). Although lower D’Amico risk groups showed an association with better continence in the univariate analysis at 1 month (*P* = .049) and 3 months (*P* = .049), this significance disappeared in the multivariable models. In the multivariate analysis at 3 months, the D’Amico risk status was not an independent predictor (*P* = .770 for low risk and *P* = .169 for intermediate risk). At 6 months, the D’Amico risk status was not a significant predictor in either the univariate (*P* = .081) or the multivariate (*P* = .749 for low risk and *P* = .417 for intermediate risk) analysis.

After 12 months, 132 patients (88.6%) were continent. Among group 2 patients, 91.9% were continent, while among group 1 patients, 85.3% were continent. Table 5 shows the distribution of patients who were continent at various times according to the study groups.

**Table 5.** Distribution of Patients with Continence at Various Times According to Study Groups

Time	All Patients	Group 1	Group 2	*P* Value^a^
First month	41 (27.5)^c^	14 (18.7)^c^	27 (36.5)^c^	**.015**
Third month	76 (51)^d^	29 (38.7)^d^	47 (63.5)^d^	**.002**
Sixth month	99 (66.4)^e^	42 (56)^e^	57 (77)^d,e^	**.007**
Twelfth month	132 (88.6)^f^	64 (85.3)^f^	68 (91.9)^e^	.208
*P* ^b^	**<.001**	**<.001**	**<.001**	

Note: The results are presented as number (%). Different lowercase letters in a column indicate a statistically significant difference between groups. Bold values indicate statistical significance (*P* < .05).

a Pearson’s chi-squared test.

b Cochran’s *Q* test.

Patients’ erectile function appears to deteriorate in the early postoperative period, but improves over time. Although the IIEF-5 scores of all patients decreased markedly in the first postoperative month, statistically significant improvements were observed in both groups over time during the 12-month follow-up period (*P* < .001). Table 6 shows the IIEF-5 total scores of patients at various time points.

**Table 6.** IIEF-5 Total Scores of Patients at Various Time Points According to Study Groups

IIEF-5 Total Score	All Patients	Group 1	Group 2	*P* Value^a^
Preoperative score	17 (12-20)^c^	17 (11-21)^c^	15.5 (12-20)^c^	.329
First month score	5 (5-7)^d^	5 (5-7)^d^	5 (5-7)^d^	.153
Third month score	6 (5-7)^d^	5 (5-7)^d,e^	7 (5-8)^d,e^	**.017**
Sixth month score	7 (5-10)^e^	6 (5-8)^e^	7 (5-10)^e^	**.030**
Twelfth month score	8 (6-11)^f^	8 (6-10)^f^	9 (6-12)^f^	.060
*P* ^b^	**<.001**	**<.001**	**<.001**	

Note: The results are presented as median (interquartile range). Different lowercase letters in a column indicate statistically significant difference between groups. Bold values indicate statistical significance (*P* < .05).

a The Mann–Whitney *U* test.

b The Friedman test.

IIEF-5, International Index of Erectile Function-5.

As a result of our study, we found a statistically significant difference in continence between the 2 groups during the first 6 months.

## Discussion

There are many publications and reviews examining incontinence after RP. The aim of this study was to examine the effect of a tube-shaped bladder neck, which we define as the modified urethral anastomosis technique, on early continence in patients undergoing RARP.

The average continence rates reported in the literature are 89%-100% at 12 months for patients treated with RARP and 80%-97% for patients treated with RRP.^16^ Similar to the literature, it was found by our study that 88.6% of patients became continent at the end of 12 months. Postoperative continence rates were 18.7%-36.5% at 1 month, 38.7%-63.5% at 3 months, 56%-77% at 6 months, and 85.3%-91.9% at 12 months for groups 1 and 2. Our new technique provided statistically superior results in the first 6 months compared with 12 months (*P* = .015, *P* = .002, *P* = .007, and *P* = .208, respectively).

A meta-analysis study by Ficarra et al examining urinary continence results after RARP found that posterior reconstruction provides a small advantage in the first month after surgery. Total anatomic reconstruction shows a significant advantage within the first 3 months.^17^ While our findings underscore the potential early continence benefits of modifying the bladder neck, it is crucial to evaluate these results within the context of alternative reconstructive techniques and opposing perspectives. Approaches that focus on restoring posterior anatomical support, such as the urethral hammock and posterior musculofascial reconstruction, have also demonstrated significant advantages in terms of early continence within the first few months.^18,19^ Furthermore, contemporary literature strongly argues that strict bladder neck preservation or local modification is not the primary determinant of postoperative urinary continence. Recent meta-analyses suggest that, although bladder neck preservation may provide some advantages in the third month, it does not substantially impact continence rates after 6 or 12 months.^20,21^ Conversely, robust evidence highlights that preserving the overall pelvic anatomy is much more critical. Advancements such as Retzius-sparing robot-assisted radical prostatectomy (RS-RARP) and single-port transvesical approaches achieve optimal and rapid early continence recovery by strictly preserving the anterior compartment, the endopelvic fascia, and the space of Retzius. This essentially leaves the bladder in its natural anatomical position.^22^ Studies on these techniques report extremely high rates of immediate continence upon catheter removal. This demonstrates that maintaining anterior structural support is more important than making isolated modifications to the bladder neck.^23^ This context emphasizes that early continence is multifactorial, and our modification serves as one of several potential strategies rather than an absolute requirement.

In a study conducted by Porpiglia et al, who performed total anatomic reconstruction in RARP, the continence rate at 6 months was reported as 98%. In the same study, the nerve-sparing technique, D’Amico risk class, LND, and prostate volume were found to be effective factors on early continence.^5^ A study investigating the pathophysiology of post-prostatectomy incontinence found that extensive anatomic dissection, neurovascular band damage, and postoperative fibrosis negatively affected continence. Bladder neck preservation and anterior reconstruction showed better continence rates.^24^

Techniques to increase urinary continence rates after RARP can be classified into 3 groups: (a) preservation (bladder neck, neurovascular bundle, puboprostatic ligament, pubovesical complex, and urethral length), (b) reconstruction (posterior/anterior reconstruction/reattachment of the arcus tendineus to the bladder neck), and (c) reinforcement (bladder neck plication and/or sling suspension).^25^

Bladder neck plication is an intervention that targets the bladder neck. Although it is not directly similar to the technique we developed, it focuses on the same goal.^26^ The ultimate goal is to narrow the bladder neck to create a functionally narrower and longer bladder neck and urethra. Our surgical suturing technique for the bladder neck is unique and has not been presented in any previous studies. However, to our knowledge, there are 3-4 studies in the literature where bladder neck plication has been performed.^26-28^

The technique most similar to ours is the controlled study conducted by Lee et al in 2011. The study included 159 bladder neck plication patients and 175 control patients, for a total of 334 patients.^26^ In this technique, 2 sutures are tied at the 3 and 9 o’clock positions, 2 cm proximal to the vesicourethral anastomosis. This gives the bladder neck a funnel shape and narrows it. According to the results of the study, the total continence rate (0 pads/d) was higher in the bladder neck plication group in the 1st and 12th months. Additionally, the time to reach total and social continence (1 pad/d) was shorter for those who underwent bladder neck plication.

In our technique, the bladder neck is narrowed from 2 points and by placing a second row of zigzag sutures at 1-cm intervals. The sutures are placed continuously until the starting point is reached again. The aim is to create a more physiological bladder neck, ensure the participation of bladder neck fibres in the anastomosis, reduce pressure on the bladder neck and sphincter, and obtain a functionally longer urethra. Continence is achieved earlier after surgery in this way. According to the number of continent patients in our study, our new technique was superior to the standard technique at the first, third, and sixth months. There is no significant difference between the 2 groups in the long term. In our study, bladder neck contracture or stricture was not observed in any patient.

A randomized, controlled study conducted by Choi et al^27^ focused on the technique applied by Lee et al. Unlike Lee’s research, this study found no statistical difference between the 2 groups in the first 6 months.

In the detrusorrhaphy technique described by Shin and Lee, bladder neck plication is performed by suturing the posterior wall of the bladder neck in a zigzag pattern.^28^ Continence rates were significantly higher in the detrusorrhaphy group than in the control group during the first 3 months. There was no significant difference found between the 6th and 12th months. The same study found no difference between the 2 groups in terms of oncological outcomes. In our study, no difference was observed between the 2 groups when surgical margin positivity was evaluated. Thus, it can be concluded that our new technique is oncologically reliable and does not compromise cancer control.

UI after RARP is one of the most important complications affecting patient comfort. Maximal preservation of the prostate, pelvic floor, vesicourethral junction, external sphincter, and adjacent anatomical structures is universally accepted as providing continence. However, the physiological mechanisms associated with urinary continence after RP are still not fully understood.^28^ We believe that many more technical modifications and studies are needed to elucidate these mechanisms, and our technique will contribute to the existing literature. The positive early continence results obtained from our study encourage us to continue this new approach.

Although the operating surgeon was highly experienced in standard RARP (>250 cases), the prolonged perioperative times and higher LND rates in group 2 reflect a technique-specific learning curve and evolving surgical scope, rather than a lack of general robotic experience. First, introducing the modified bladder neck technique required meticulous double-row suturing, naturally prolonging the median anastomosis time (30 vs 20 minutes), and contemporary literature confirms that novel reconstructive modifications initially reset the learning curve until a consolidation phase is reached.^29^ Second, the longer overall operative times in the later cohort were primarily driven by increased institutional adherence to ePLND guidelines (51.4% vs 33.3%), a procedure well documented to significantly increase total console time and surgical complexity.^30^ Consequently, the higher rate of minor intraoperative organ injuries observed in group 2 (*P* = .013) was primarily due to the anatomical challenges and larger dissection areas required for these ePLND procedures.

Our study has several limitations, including a small sample size and a single-centre design. One limitation was the imbalance in baseline characteristics between the HT and ED subgroups. Nevertheless, total IIEF scores were comparable between groups, and our multivariable analysis revealed that the modified technique was the primary factor in continence recovery, even when accounting for these comorbidities. Data on smoking habits, a known potential confounding factor for urinary continence recovery, were unavailable for analysis due to the retrospective nature of the study. Due to the modest sample size within specific stratifications, such as the ‘high-risk D’Amico’ group, we prioritized multivariable logistic regression analysis over direct subgroup comparisons. This methodological approach allowed us to adjust for potential confounders across the entire cohort, thereby maximizing statistical robustness and integrity. Additionally, our study did not objectively evaluate certain anatomical confounding factors. Specifically, preoperative membranous urethral length, a known predictor of continence, was not systematically assessed using magnetic resonance imaging. Furthermore, given the sequential nature of the study, it cannot be ruled out that the surgeon’s ongoing learning curve and accumulated experience contributed to the superior outcomes favouring the modified group, as it was not possible to statistically isolate these continuous variables in our analysis.

The modified urethral anastomosis technique accelerates the return of urinary continence in the early postoperative period and is comparable to the standard approach in terms of long-term efficacy. Our continence rates are comparable to those reported in the literature. Our new technique is oncologically reliable and does not compromise cancer control. However, more randomized, controlled, prospective, and multicentre studies are needed to clarify the current evidence regarding this new approach.
